# Evidence for frozen melts in the mid-lithosphere detected from active-source seismic data

**DOI:** 10.1038/s41598-017-16047-4

**Published:** 2017-11-17

**Authors:** Akane Ohira, Shuichi Kodaira, Yasuyuki Nakamura, Gou Fujie, Ryuta Arai, Seiichi Miura

**Affiliations:** 1 Yokohama National University, Tokiwadai 79-1, Hodogaya-ku, Yokohama, 240-8501 Japan; 2 R&D Center for Earthquake and Tsunami (CEAT), Japan Agency for Marine-Earth Science and Technology (JAMSTEC), Showa-machi 3173-25, Kanazawa-ku, Yokohama, 236-0001 Japan

## Abstract

The interactions of the lithospheric plates that form the Earth’s outer shell provide much of the evidentiary basis for modern plate tectonic theory. Seismic discontinuities in the lithosphere arising from mantle convection and plate motion provide constraints on the physical and chemical properties of the mantle that contribute to the processes of formation and evolution of tectonic plates. Seismological studies during the past two decades have detected seismic discontinuities within the oceanic lithosphere in addition to that at the lithosphere–asthenosphere boundary (LAB). However, the depth, distribution, and physical properties of these discontinuities are not well constrained, which makes it difficult to use seismological data to examine their origin. Here we present new active-source seismic data acquired along a 1,130 km profile across an old Pacific plate (148–128 Ma) that show oceanic mid-lithosphere discontinuities (oceanic MLDs) distributed 37–59 km below the seafloor. The presence of the oceanic MLDs suggests that frozen melts that accumulated at past LABs have been preserved as low-velocity layers within the current mature lithosphere. These observations show that long-offset, high-frequency, active-source seismic data can be used to image mid-lithospheric structure, which is fundamental to understanding the formation and evolution of tectonic plates.

## Introduction

Previous seismological observations have identified negative seismic velocity discontinuities at 35–120 km depth in the upper mantle; these are collectively known as the Gutenberg discontinuity (G discontinuity)^1^. Many studies have interpreted the G discontinuity to represent the LAB because both features are observed at similar depths. However, some studies have indicated that the G discontinuity cannot be explained by thermal structure alone and that there are other seismic discontinuities within the shallow upper mantle that cannot be interpreted to represent the LAB^1–6^. For instance, a sharp decrease in seismic velocity (~6%) has been imaged at ~60–70 km depths in the lithosphere under the central Pacific^2–4^. This discontinuity has been variously attributed to a compositional boundary formed during melt extraction at mid-ocean ridges^3,7,8^, to a change in water content in the lithosphere^9^, to seismic anisotropy in the lithosphere^6,10,11^, or to the presence of partial melt in the lithosphere^4^. Seismic data at the depths of such lithospheric discontinuities might reveal information about the processes of formation and evolution of tectonic plates. However, there are several unresolved issues related to the use of seismic data for this purpose, three of which we describe briefly below. (1) Although multibounce S waves (SS waves) and reverberations of S waves reflected from the core–mantle boundary (ScS waves) have shown sharp negative velocity discontinuities of ~30 km thickness at a constant depth under the Pacific plate^3,4^, they cannot resolve the distribution of such sharp discontinuities because of their long wavelength. Moreover, the use of SS precursors to detect shallow (<50 km depth) discontinuities is difficult^12^. (2) Receiver function analysis using converted waves has imaged sharp boundaries with an effective resolution for a layer thickness of 10 km^5^. However, several studies have pointed out that these observations were limited to the vicinity of ocean islands on which seismic stations were deployed^1,5^. Consequently, little information is available about the G discontinuity in regions of simple tectonics that would be better suited to investigation of the evolutionary processes of the lithosphere. (3) Although an active-source seismic study has detected reflections from the deep mantle under a ~120-Myr-old Pacific plate^13^, there is a paucity of data from which to establish the presence (or absence) of the G discontinuity within the older parts (>130 Ma) of tectonic plates^1,6^ where evidence is likely preserved of ancient tectonic processes that are important for investigations of the origin of the G discontinuity.

**Figure 1:**
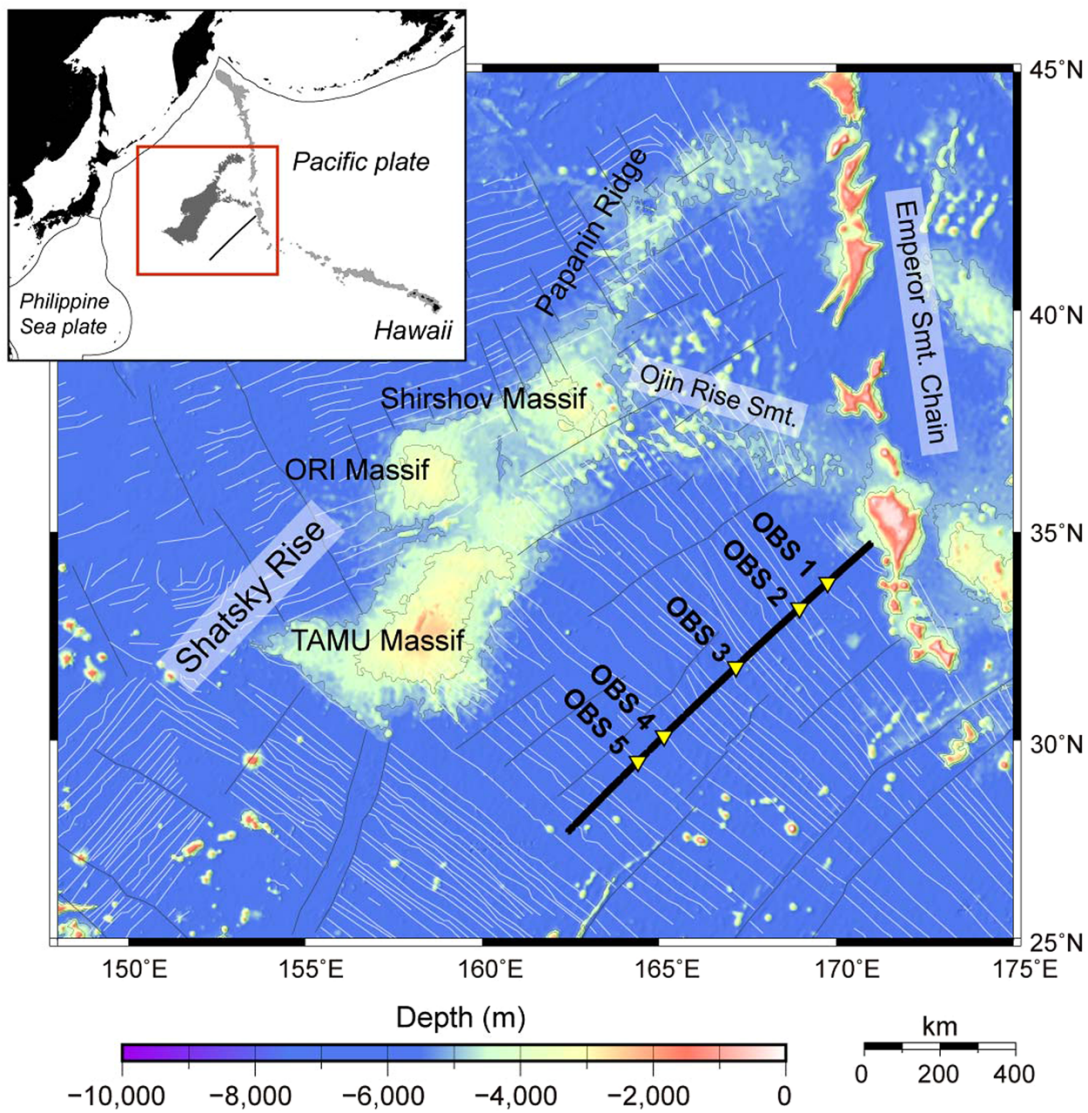
Regional and local location maps of the study area. The black line marks the location of the seismic refraction and reflection survey profile. An airgun array with a total volume of 7,800 cubic inches was fired at intervals of 200 m along the line. Yellow triangles indicate the position of ocean bottom seismometers (OBSs 1–5). The survey line is southeast of the Shatsky Rise on the Pacific plate (inset map). Fine white lines on the main panel are magnetic lineations^15^. Generic Mapping Tools 5 (https://www.soest.hawaii.edu/gmt/) was used to draw figures.

In 2014, we recorded active-source seismic reflection and refraction data along a 1,130 km profile across a part of the Pacific plate that formed during 148–128 Ma, southeast of the Shatsky Rise in the northwestern Pacific (Fig. 1 and Methods). Because the original objective of that survey was to record reflections from the Moho by hydrophones in a towed streamer^14^, we deployed only five ocean bottom seismometers (OBSs) from which to obtain supplementary information about crustal velocity structure. However, by recording long-offset data, we observed unexpected deep reflections on the OBS records, which are the subject of this paper. Note that this study was designed as a 2-D seismic survey, so we analyzed our data assuming a 2-D velocity structure.

The Pacific plate in this region was formed at the fast-spreading Pacific–Farallon ridge (half spreading rate of ~40 mm yr^−1^)^15^. Our profile was oriented perpendicular to northwest-trending magnetic anomalies M20 to M1 (spanning seafloor ages of 148–128 Ma^16^) and did not cross any fracture zones. Thus, our seismic profile crossed a tectonically simple ocean basin on the old Pacific plate and may address some of the issues (discussed above) that impede seismic investigations of the processes of formation and evolution of oceanic lithosphere.

## Results

**Figure 2:**
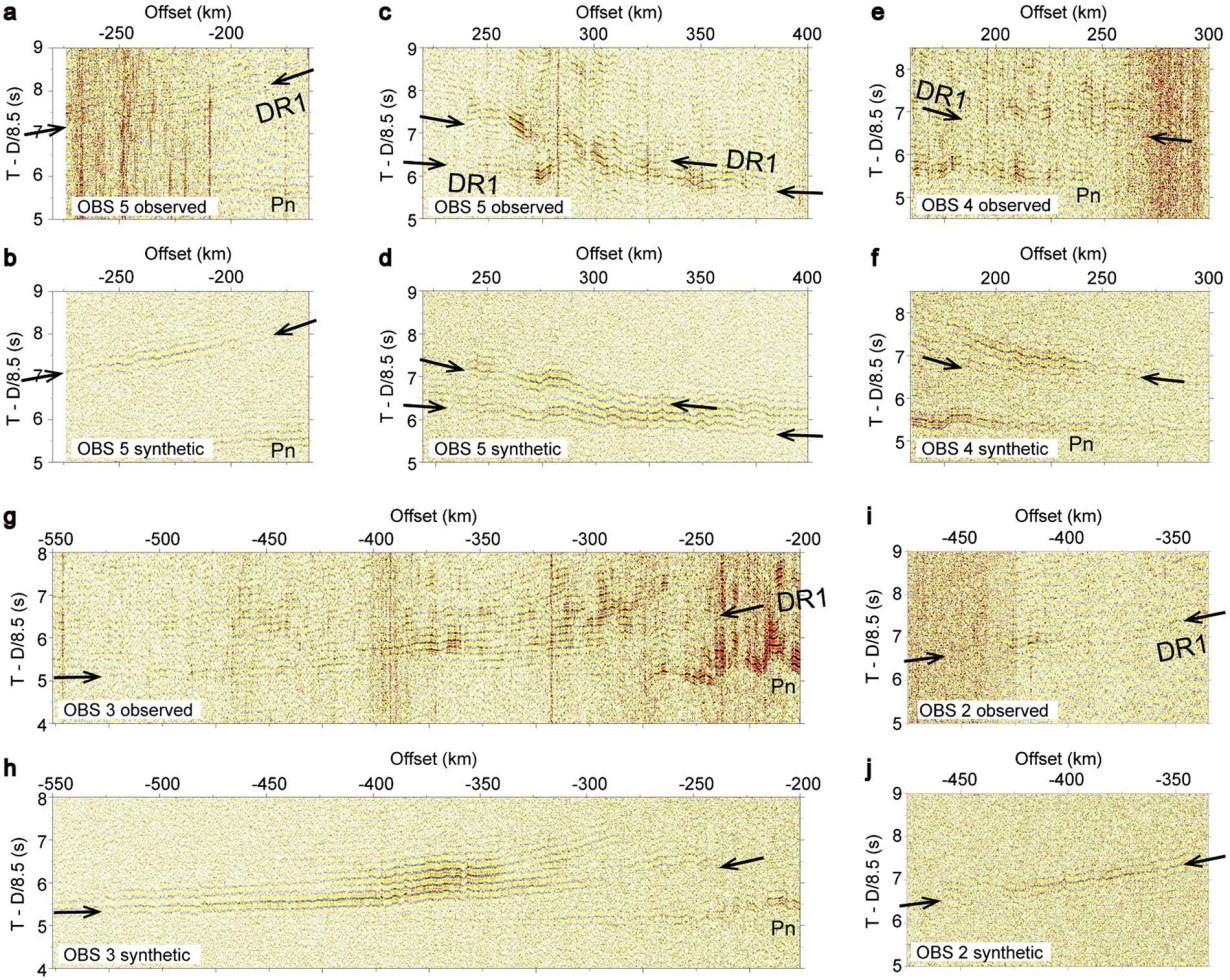
Observed and synthetic waveforms for OBSs 2–5. DR1 (arrows) indicates wide-angle reflection phases from the oceanic MLDs. Pn indicates refraction phases from the uppermost mantle. Observed and synthetic waveforms were band-pass filtered (5–20 Hz). Vertical axes were reduced using a velocity of 8.5 km s^−1^. The 900 km offset range of records from OBSs 2–5 are shown in Supplementary Fig. S1. Observed and synthetic waveforms show reflections from the oceanic MLD at distance of (**a**,**b**) 140–190 km (Fig. 3a), (**c**,**d**) 395–440 km and 400–460 km, (**e**,**f**) 465–490 km, (**g**,**h**) 410–495 km, and (**i**,**j**) 660–695 km.

Here we summarize the results of our previous analysis of crustal velocity structure and multichannel seismic (MCS) sections^14^. The velocity structure we determined is typical of old oceanic crust^17^. It should be noted that the P-wave velocity of the uppermost mantle, which is well constrained by clear refraction arrivals (Pn in Fig. 2), is remarkably high (up to 8.6 km s^−1^). This high velocity suggests that strong azimuthal anisotropy along the fossil spreading direction, owing to the alignment of olivine crystals, has been preserved in the mantle^18,19^. On the MCS sections^14^, the character of the Moho reflections varied along the profile, even though the spacing between anomalies M20 and M1 indicates that the rate of seafloor spreading changed little during the corresponding time period. We observed clear reflections from the Moho at the southwestern end of the profile, but they were diffuse, weak, or absent along the rest of the profile. Because the character of the Moho reflections changed notably immediately following the initial stage of formation of the Shatsky Rise, we interpreted the thick crust–mantle transition layer we observed to be the result of post-magmatic igneous activity that occurred some time after a crustal accretion along the mid-oceanic ridge.

Common features we observed on records from OBSs 2 to 5 were wide-angle reflection phases at offsets up to about 500 km (DR1 hereafter). These phases were not recorded at OBS 1 because of low signal-noise ratio. Although the reflection signals are weak, we identified hyperbolic arrivals at 5–8 s (reduction velocity of 8.5 km s^−1^) at offsets from 180 to 525 km on each OBS record (Fig. 2).

**Figure 3:**
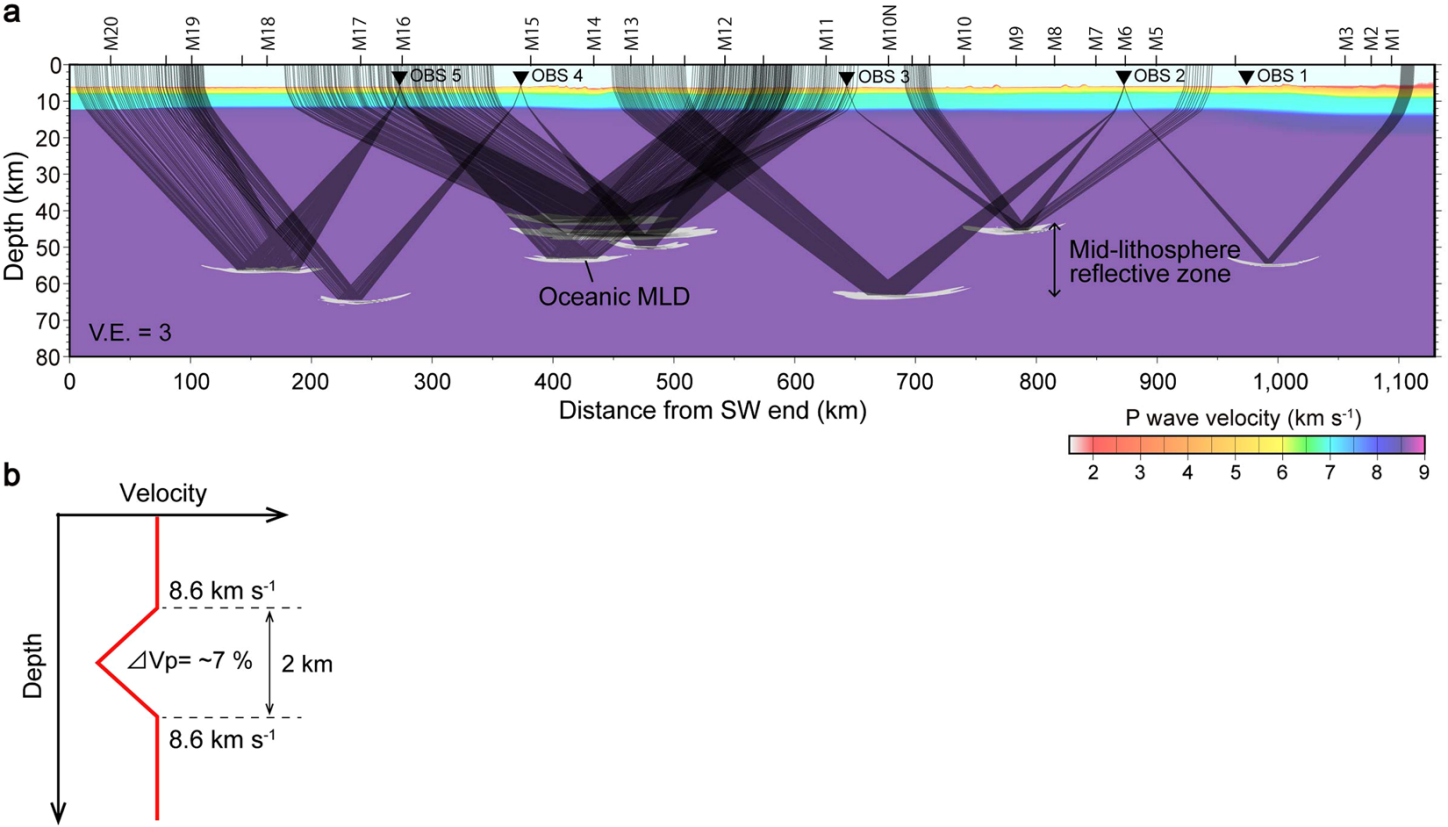
Results of traveltime analyses and preferred velocity model for oceanic MLD. (**a**) Ray tracing based on traveltime picks of DR1. The background velocity model was used to determine reflection traveltime picks. The traveltime picks and calculated arrivals are shown in Supplementary Fig. S1. Magnetic anomaly numbers (M20–M1) are shown along the top of the profile. (**b**) P-wave velocity model for each reflector (oceanic MLD) with a gradual velocity change (maximum ~7% decrease). Based on our ray tracing, we placed the low-velocity structure on observed reflection points, and then calculated synthetic waveforms (Fig. 2). Waveform calculation tests with other velocity models are shown in Supplementary Figs S2 and S3.

Using 2-D traveltime analysis, we estimated the reflection depth of the DR1 to be 37 to 59 km below the seafloor (within the mid-lithosphere reflective zone of Fig. 3a; see Methods) by assuming a constant mantle velocity above the reflectors^20^. We refer to these reflectors as oceanic MLDs. We also considered the possibility that the reflectors that produced DR1 were a considerable horizontal distance from the profile (i.e., they represent sideswipe), in which case they must dip steeply. It is difficult to formulate a plausible geologic explanation for the existence of such reflectors in the tectonic setting of our survey area. Nonetheless, to further investigate the possibility that DR1 reflectors represent sideswipe, we rotated the two horizontal components into radial and transverse components, then analyzed the polarization (see Methods). The results of this analysis confirmed that the particle motions support our view that the DR1 reflections are unlikely to include a substantial component of sideswipe energy. If DR1 reflections did include a substantial sideswipe component, we would expect to observe clearly transverse particle motion in the horizontal components. However, the observed horizontal particle motion of DR1 reflections was similar to and of comparable amplitude to that of the background noise (Supplementary Fig. S7).

Based on the results of the traveltime analysis, we calculated synthetic waveforms by using finite-difference elastic wave propagation modeling^21^ and estimated the velocity structure required to generate the observed waveforms (see Methods). Because of the wide OBS spacing along our profile, we used a forward modeling approach to examine four possible velocity structures for the oceanic MLDs: discrete low-velocity layers (~50 km lateral extent) with gradual vertical changes in velocity (Supplementary Fig. S2a), the same low-velocity layers but with abrupt velocity changes (Supplementary Fig. S2b), discrete high-velocity layers with gradual velocity changes (Supplementary Fig. S2c), and horizontally continuous low-velocity layers with gradual velocity changes (Supplementary Fig. S2d). We assumed the maximum change of velocity to be ~7%^1,2^ and the anomalous velocity layers to be 2 km thick, which is the minimum thickness of an anomalous layer that would produce the reflection phases observed in this study.

The model with discrete low-velocity layers with gradual velocity changes (Fig. 3b) provided the best match with the observed waveforms with respect to the first arrivals, offset ranges, traveltimes, and amplitudes of the DR1 (Fig. 2). The other velocity models (Supplementary Fig. S2b–d) did not provide an explanation of the observed waveforms; in fact, they produced narrow angle synthetic reflections (arrows in Supplementary Fig. S3) at shorter offsets than the observed DR1 that were not evident in the observed records. Because we used a forward modeling approach, we did not obtain a unique solution; however, only the model shown in Fig. 3b provided a plausible reproduction of the observed OBS records. Reverberations recorded after the first arrivals of DR1 (Fig. 2) suggest that there might be more oceanic MLDs, possibly arranged in a vertically stacked laminated structure. Moreover, because of the sparse deployment of OBSs, the ray paths of the seismic energy we recorded do not sample all of the mid-lithosphere along the survey profile. Consequently, there may also be more oceanic MLDs distributed laterally along the profile.

Another remarkable observation was a wide-angle reflection phase recorded at OBS 2 at offsets up to at least ~850 km (Supplementary Fig. S4a; DR2 hereafter). The reflection depth of DR2 depends on the velocity structure within the mantle, which is difficult to estimate from our data, but by assuming a P-wave velocity in the mantle of 8.3 km s^–1^ below the mid-lithosphere reflective zone (Model B in Supplementary Fig. S4b), we calculated the reflection depth of DR2 to be 108 km (102 km beneath the seafloor). It is noteworthy that DR2 differs from DR1 in that it is observed continuously over a wide range of offsets (more than 300 km) and that the reflector that produced it is ~170 km long (Supplementary Fig. S5). In addition, DR2 is a much weaker signal than DR1. We interpreted DR2 to be a reflection from the LAB, which has been observed as an age-dependent discontinuity in previous studies^12,22–25^.

## Discussion

Our high-frequency active-source seismic data imaged oceanic MLDs at depths of 37–59 km, which we call the mid-lithosphere reflective zone, and also imaged the LAB. The depths of the oceanic MLDs are similar to those of negative velocity discontinuities in the shallow upper mantle observed by previous studies^1–4,26^. For instance, studies using SS precursors have imaged the G discontinuity below the Pacific plate at 40–100 km depth, though the number of samples in the old plate (>120 Ma) was limited in those studies. It has also been suggested that reflectivity at the G discontinuity is enhanced in tectonic settings where melts produced by post-magmatic activity in the lithosphere^1^. More recent studies of SS precursors have imaged similar discontinuities at 43–80 km depth under the Pacific Ocean; these have no age dependence in lithosphere older than 36 Ma and have been interpreted to represent compositional discontinuities due to melts frozen within the lithosphere^26^. In addition to seismological observations, experimental studies^27–30^ have shown that mobile melts trapped at the top of the asthenosphere have been incorporated into the cooling lithosphere and remain there as frozen melts. Therefore, we interpreted DR1 to represent an age-independent compositional interface. Our interpretation of DR2 as a reflection from the LAB is supported by another active-source seismic survey profile that imaged reflections from the LAB beneath the subducting Pacific plate^13^, although that survey differed from ours in its tectonic setting, seismic energy source, and reflection angles.

We propose that the oceanic MLDs represent melts that were produced by post-magmatic activity after the formation of crust at a mid-ocean ridge and were then accreted and frozen at the LAB that existed at the time, which is shallower than the current LAB. This interpretation places the mid-lithosphere reflective zone at a depth (37–59 km) that corresponds to the depth of the LAB below the seafloor 10–30 million years after the initial formation of new crust at the spreading ridge. The modified Moho structure we identified on our MCS section supports this interpretation of post-magmatic activity^14^. An alternative explanation of oceanic MLDs is that grain-boundary sliding due to hydration caused the sharp seismic discontinuity at ~70 km depth in the old oceanic plate^9^. Hydration may also contribute to the sharp velocity decrease that we observed, but further examination is needed of the effect of hydration on the oceanic MLDs that we observed because they are shallower than the seismic discontinuity (~70 km) used to advance the hydration theory.

**Figure 4:**
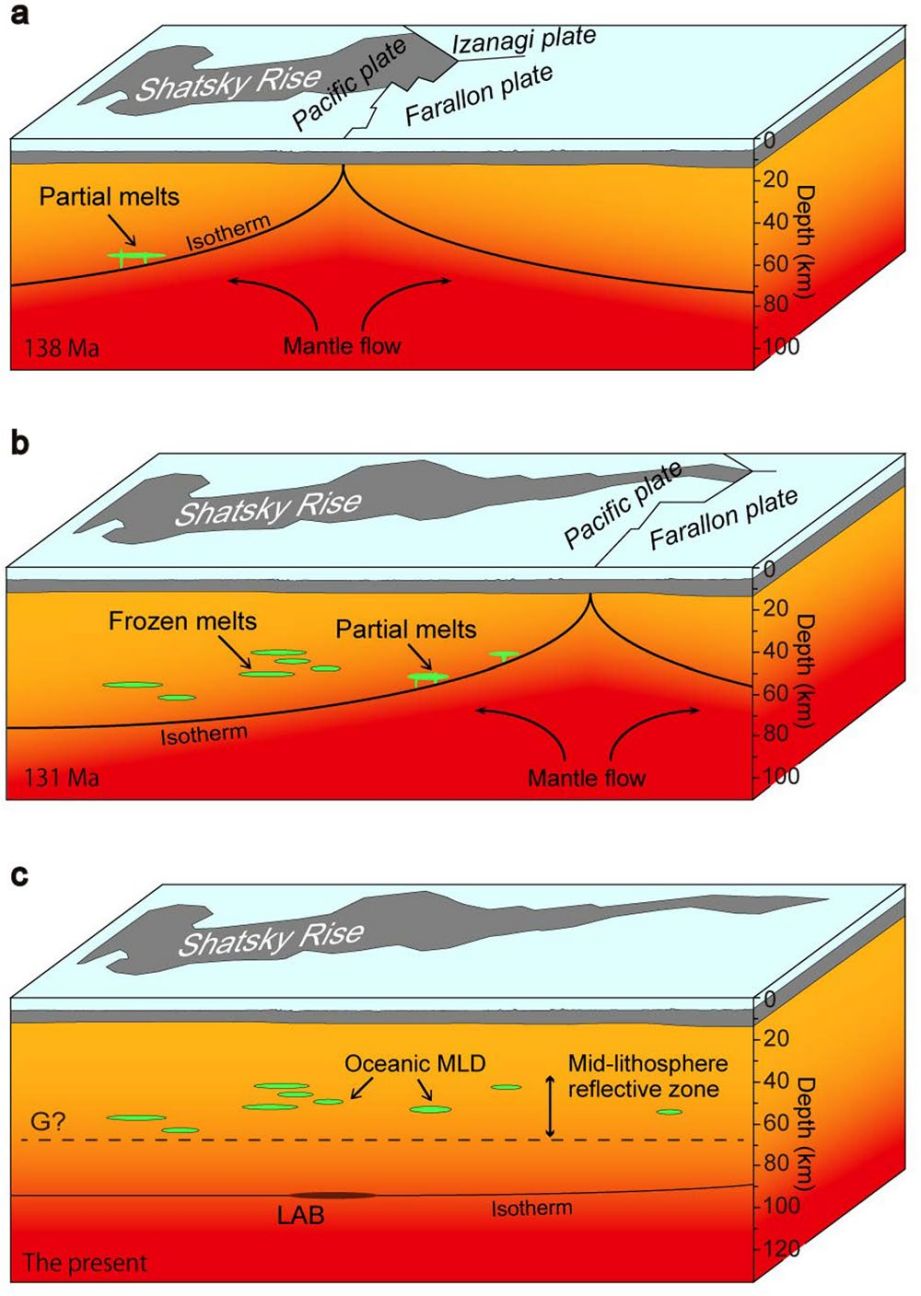
Schematic model for formation of oceanic MLDs. (**a**) Formation and emplacement of a melt at the lithosphere–asthenosphere boundary (LAB) on the flank of a mid-ocean ridge. The black line (isotherm) indicates the position of the LAB. Ascending mantle flow causes shallow decompression and melting with extracted melts ponded at and immediately above the LAB. (**b**) Formation of frozen melts incorporated in the lithosphere. As the lithosphere thickens away from the ridge, the melts are aligned horizontally in response to shear deformation as the plate moves away from the ridge and the melts freeze within the cooling lithosphere. (**c**) Present-day lithosphere preserving frozen melts as oceanic MLDs within the mid-lithosphere reflective zone. The dashed line indicates the approximate depth of the G discontinuity inferred from the results of previous studies^2,22–25^. The black shadow on the isotherm indicates the reflection position of the DR2 (Supplementary Fig. S4), which we interpreted to represent a thermal structure at the present-day LAB.

In the following we propose a model to explain the geodynamic development of the oceanic MLDs based on their distribution and the results of previous petrological studies (Fig. 4). Ascending convectional mantle flow causes shallow decompression melting beneath the mid-ocean ridge. The resultant volatile-rich partial melts impinge on the LAB and accumulate at and immediately above the boundary^27,28,30^. The trapped melts are then aligned horizontally in response to shear deformation as new crust forms and moves away from the ridge (Fig. 4a). As the plate thickens away from the ridge, the melts are incorporated and frozen in the cooling lithosphere because the LAB acts as a freezing front^27,29,31^ (Fig. 4b). The mature (present-day) lithosphere (Fig. 4c) preserves the frozen melts that were incorporated into the mid-lithosphere reflective zone. A detailed discussion of the composition of the frozen melts is beyond the scope of our study, but a typical residual mantle (i.e., harzburgite) and orthopyroxene-poor peridotite (i.e., wehrlite), which is formed by crystallization of MORB-type melts^32^, may explain the ~6% of velocity decrease we observed^33^.

Quasi-laminated structures similar to those we identified and related to frozen melts within oceanic lithosphere have been identified from Po and So phases, which are characterized by high-frequency content and great distance of travel through the oceanic lithosphere^34^. Whether such frozen melts are ubiquitous in the lithosphere or restricted to specific regions is beyond the scope of our study. However, our identification of oceanic MLDs in active-source seismic data indicates that similar long-offset active-source profiling can be used to image deep lithospheric structure and that further regional and global multi-scale studies have the potential to improve our understanding of the evolution of tectonic plates.

## Methods

### Data acquisition

Five ocean bottom seismometers (OBSs) were deployed and recovered by R/V *Kairei*. An airgun array (volume 7,800 cubic inches) was fired at 200 m intervals along the profile. In addition to seismic refraction data, multi-channel seismic reflection data were collected with a 444-channel, 6,000-m streamer cable.

### Traveltime analysis

Depths below seafloor of oceanic MLDs were estimated using traveltime picks of the DR1. For traveltime mapping^35^, we projected reflection traveltime picks in the time–distance domain into the depth–distance domain by adopting a P-wave velocity structure model^14^ estimated by applying conventional traveltime analysis^36^ for the crust and uppermost mantle. For the deeper part of the lithosphere where Pn waves do not penetrate, we extrapolated the velocity of the uppermost mantle (Vp = 8.6 km s^−1^) assuming it to be an anisotropic layer^20^ (Fig. 3a). We then determined the actual reflection point by using two-dimensional ray-tracing^36^ and calculated the theoretical traveltimes of DR1 to each OBS (Supplementary Fig. S1).

### Model uncertainties

The uncertainties of picked two-way reflection arrival times were ±0.3 s (Supplementary Fig. S1). The uncertainties of reflection depths were calculated by multiplying the one-way traveltime uncertainties (±0.15 s) by the average velocity above the reflector^37^. In this study, the errors in the depths of reflectors were ±1.0 to 1.1 km based on an average velocity above the reflectors of 6.9–7.5 km s^−1^.

### Calculation of waveforms

To calculate the synthetic waveforms (Fig. 2, Supplementary Figs S1 and S3) we used a finite-difference elastic wave propagation algorithm^21^ in which wave equations are solved on a staggered grid^38–40^. The finite-difference approximation is second order in time and fourth order in space, with a grid spacing of 30 m and a time interval of 1 ms. We calculated the synthetic waveforms based on the P-wave velocity structures shown in Supplementary Fig. S2. The density (ρ) was calculated as

$${\rm{\rho }}=-0.6997+2.2302{\rm{Vp}}-0.598{{\rm{Vp}}}^{2}+0.07036{{\rm{Vp}}}^{3}-0.0028311{{\rm{Vp}}}^{4}$$

where Vp is P-wave velocity^41^. Seismic attenuation quality factors for both P waves (Qp) and S waves (Qs) were included in the waveform calculation as follows: Qp = 50 and Qs = 10 in the sediment layer (Vp < 2.5 km s^−1^); Qp = ~500 and Qs = ~250 in oceanic crust (2.5 ≤ Vp < 7.6 km s^−1^); and Qp = 1000, Qs = 500 in the mantle (Vp ≥ 7.6 km s^−1^). Vp/Vs in the mantle was assumed to be 1.73. For the source–time function we used the observed source wavelet that was recorded during firing of the airgun array of R/V *Kairei*. For the synthetic waveforms we added white noise (5–15 Hz) to each trace. The amplitudes of the synthetic waveforms were divided by r^0.5^, where r is offset, to correct for the difference of geometrical spreading effects for the 2-D calculation and the actual 3-D wavefield.

### Polarization analysis

To investigate whether or not DR1 represent sideswipe, we analyzed the two horizontal components of the data from each OBS record. First, we used the polarization of the direct water waves of airgun shots to determine the radial and transverse components of the seismic energy. The observed waveforms of the radial and transverse components corresponding to those shown in Fig. 2 are shown in Supplementary Fig. S6. The transverse components hardly have any remarkable phase on the each section, which suggests that the reflections are from below the seismic profile rather than from the side. Then we plotted particle motions in the radial–horizontal plane for a duration of 1 s starting 0.3 s before the first arrival times of each DR1, and compared them with those of a 1 s duration of background noise (Supplementary Fig. S7). Particle motions plotted at offset intervals of 10 ± 1 km (indicated by arrows in Supplementary Fig. S6) show that the particle motions of the DR1 are much the same as those of the background noise, which makes it difficult to interpret the DR1 as sideswipe.

### Data Availability

The datasets generated and analyzed during this study are available from the JAMSTEC data-base site at https://www.jamstec.go.jp/jamstec-e/IFREE_center/index-e.html.

## Electronic supplementary material

Supplementary Information
