# Supplementary material for: Evidence for frozen melts in the mid-lithosphere detected from active-source seismic data
Source: Sci Rep. 2017 Nov 17;7:15770. doi: 10.1038/s41598-017-16047-4 (PMC5693938; doi:10.1038/s41598-017-16047-4)
Supplement: Supplementary file 1 — Supplementary Information [file 41598_2017_16047_MOESM1_ESM.pdf]

Supplementary Information

Evidence for frozen melts in the mid-lithosphere detected from active-source seismic data

Akane Ohira[1,2]*, Shuichi Kodaira[1,2], Yasuyuki Nakamura[2], Gou Fujie[2], Ryuta Arai[2], Seiichi Miura[2]

[1] Yokohama National University, Tokiwadai 79-1, Hodogaya-ku, Yokohama 240-8501, Japan

[2] R&D Center for Earthquake and Tsunami (CEAT), Japan Agency for Marine-Earth Science and Technology (JAMSTEC), Showa-machi 3173-25, Kanazawa-ku, Yokohama 236-0001, Japan

*corresponding author (A. Ohira), E-mail: ohira@jamstec.go.jp

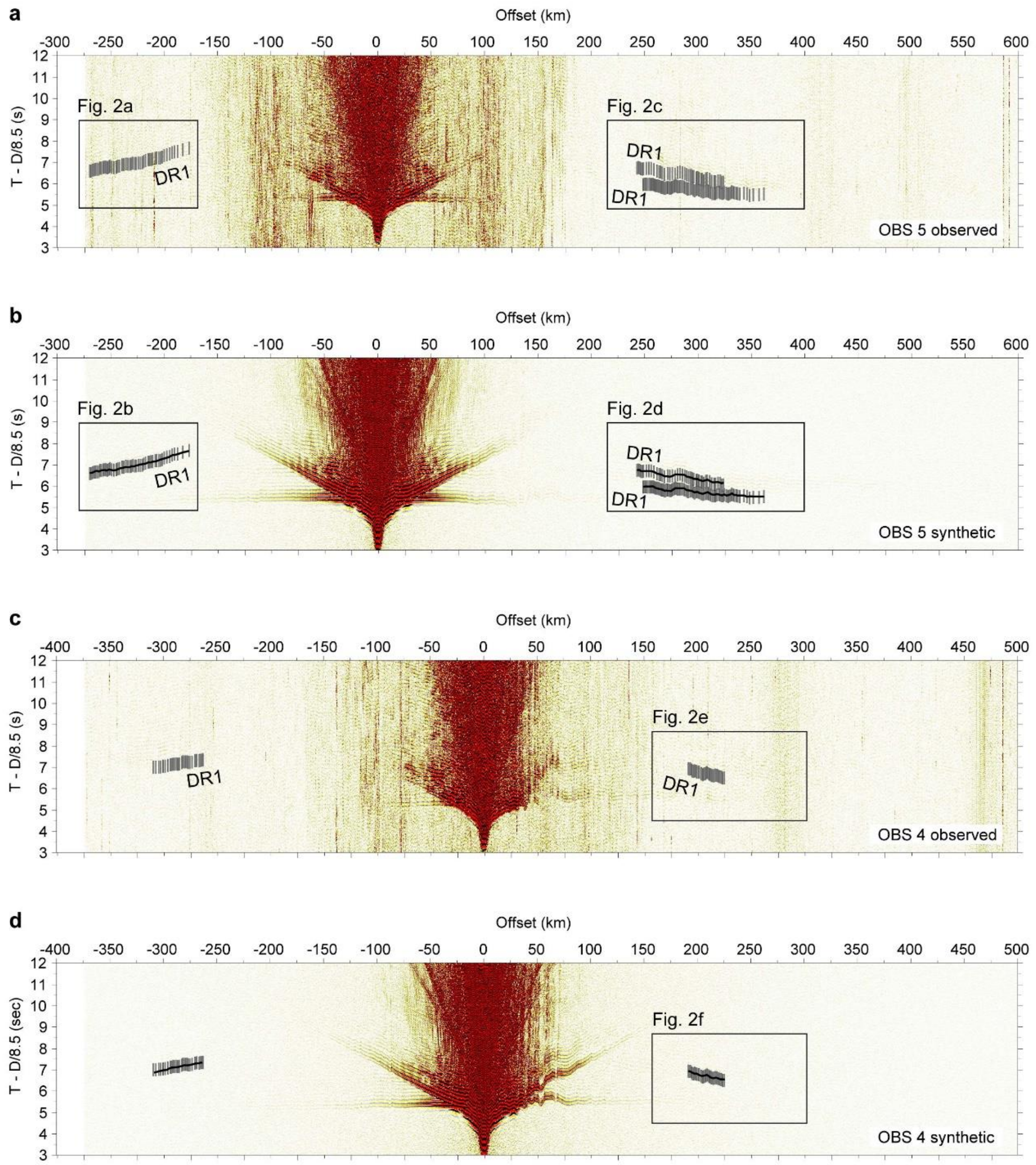
a
Offset (km)
T - D/8.5 (s)
Fig. 2a
DR1
Fig. 2c
DR1
DR1
OBS 5 observed
b
Offset (km)
T - D/8.5 (s)
Fig. 2b
DR1
Fig. 2d
DR1
DR1
OBS 5 synthetic
c
Offset (km)
T - D/8.5 (s)
DR1
Fig. 2e
DR1
OBS 4 observed
d
Offset (km)
T - D/8.5 (sec)
Fig. 2f
OBS 4 synthetic

19

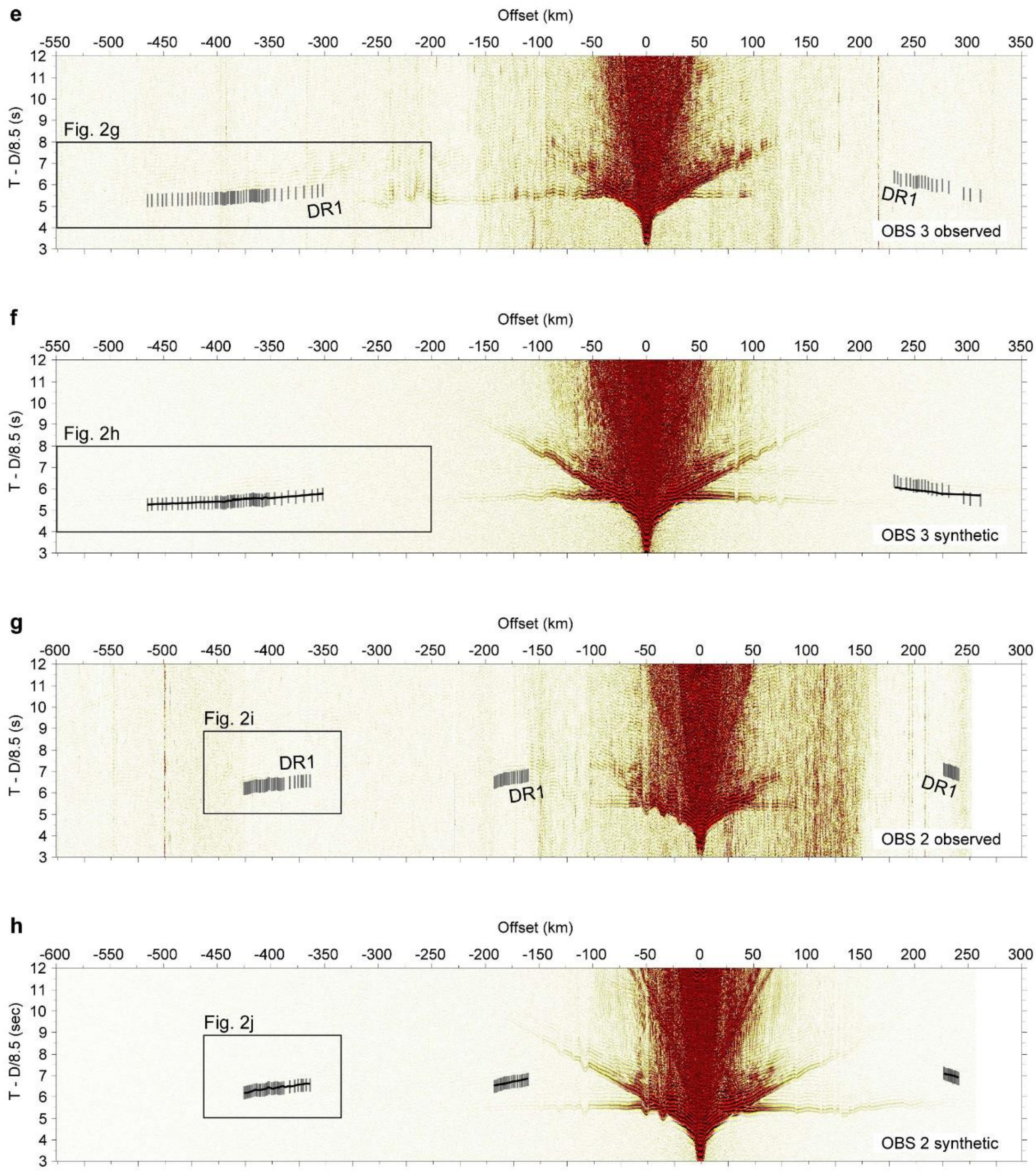
e
Offset (km)
T - D/8.5 (s)
Fig. 2g
DR1
DR1
OBS 3 observed
f
Offset (km)
T - D/8.5 (s)
Fig. 2h
OBS 3 synthetic
g
Offset (km)
T - D/8.5 (s)
Fig. 2i
DR1
DR1
DR1
OBS 2 observed
h
Offset (km)
T - D/8.5 (sec)
Fig. 2j
OBS 2 synthetic

Supplementary Figure S1. Observed and synthetic waveforms over 900 km offset ranges for OBSs 2–5. DR1 indicates wide-angle reflection phases from the oceanic MLD. Both observed and synthetic waveforms were band-pass filtered (5–20 Hz). Vertical axes were reduced using a velocity of 8.5 km s^{-1}. Vertical bars on the records show traveltime picks for DR1. Black lines on the synthetic waveforms show calculated traveltimes based on ray tracing. (**a**–**b**) OBS 5; (**c**–**d**) OBS 4; (**e**–**f**) OBS 3; (**g**–**h**) OBS 2.

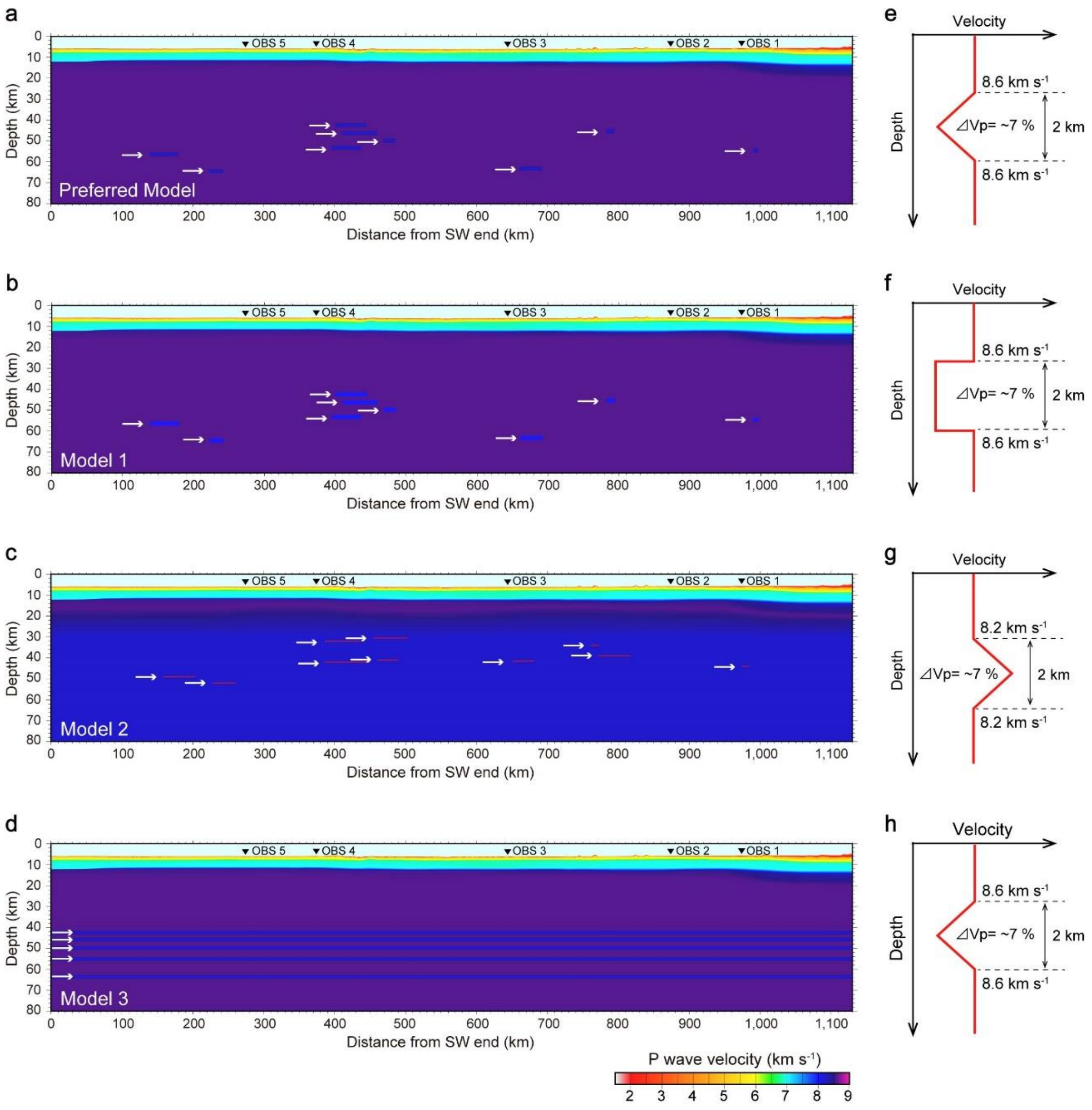

Supplementary Figure S2. Application of four P-wave velocity models for waveform calculations of DR1. White arrows point to the reflectors representing oceanic MLDs. (**a**) Preferred model with reflections from discrete low-velocity layers with gradual changes of velocity over 2 km depth ranges. (**b**) Model 1 with reflections from discrete low-velocity layers with abrupt velocity changes. (**c**) Model 2 with reflections from high-velocity layers with gradual velocity changes. (**d**) Model 3 with reflections from

horizontally continuous low-velocity layers with gradual velocity changes. P-wave velocity models for panels **a–d** are shown in panels **e–h**. The results of this modeling are shown in OBS record format in Fig. 2 and Supplementary Fig. S3.

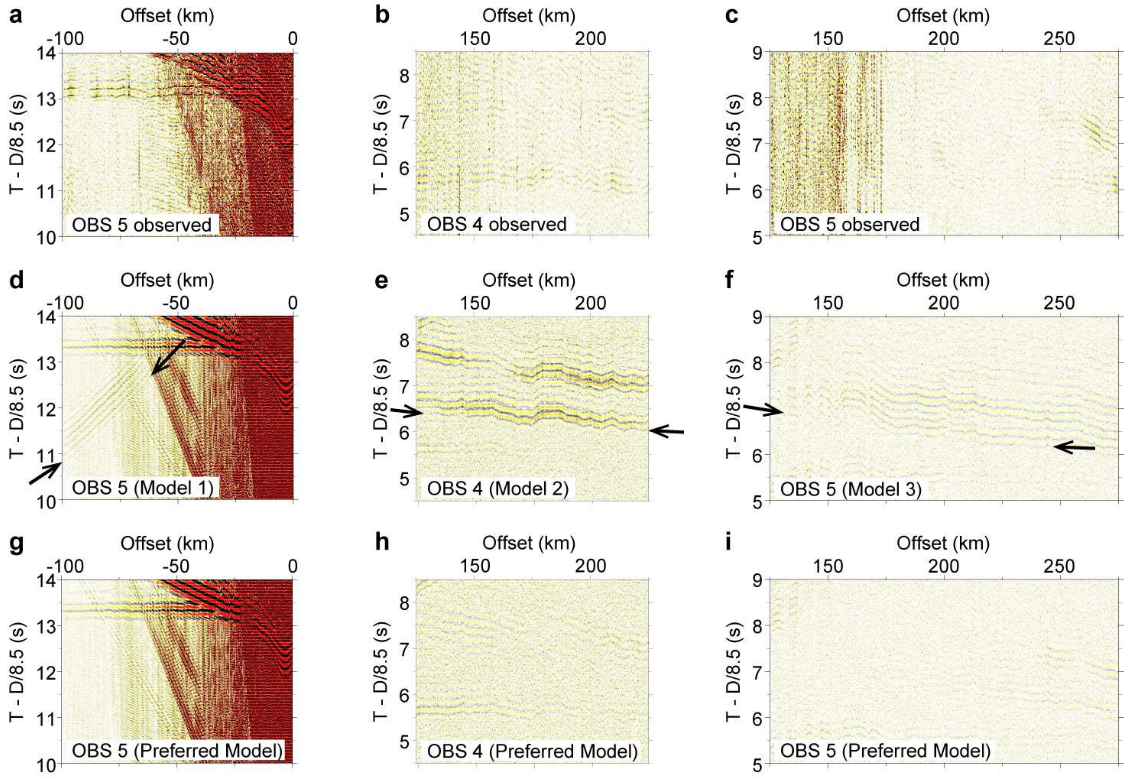

Supplementary Figure S3. Examples of results of waveform modeling for DR1. (**a**–**c**) Observed waveforms for OBS 4 and OBS 5 (two offset ranges) and (**d**–**f**) corresponding synthetic waveforms calculated with models 1, 2, and 3, respectively (Supplementary Fig. S2b–d). The black arrows in panels **d**–**f** point to phases that were not present in the observed waveforms, indicating that models 1–3 did not reproduce the observed records. **g**–**i**, Synthetic waveforms for the same OBSs as above but calculated with the preferred model (Supplementary Fig. S2a).

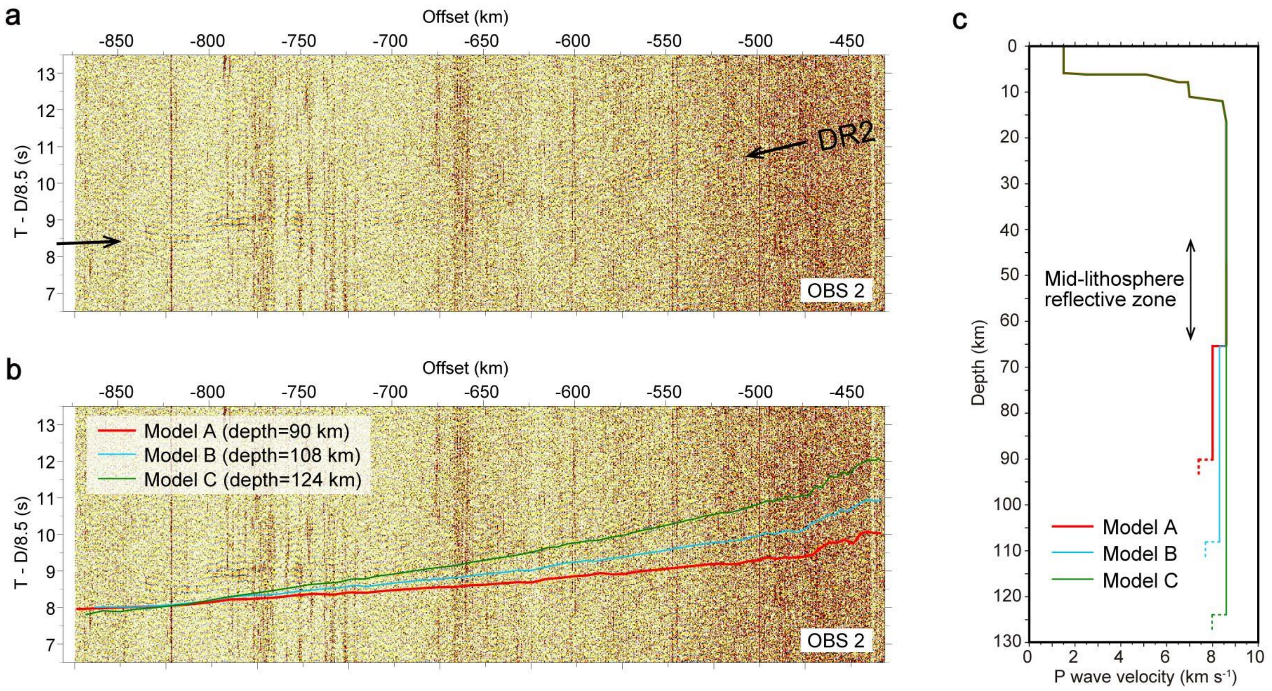

Supplementary Figure S4. Reflection depth of DR2 according to three assumed P-wave velocities in the mantle below the mid-lithosphere reflective zone. (**a**) Waveforms at large offsets observed at OBS 2. Arrows point to the DR2. (**b**) Calculated traveltimes superimposed on the observed record. Traveltime curves of reflection phases from 90 km depth (red), 108 km depth (cyan), and 124 km depth (green) according to 1-D P-wave velocity models A, B, and C, respectively, shown in (**c**). The base of the lithosphere in each of the velocity models is represented by a sharp velocity decrease according to refs. 13 and 24.

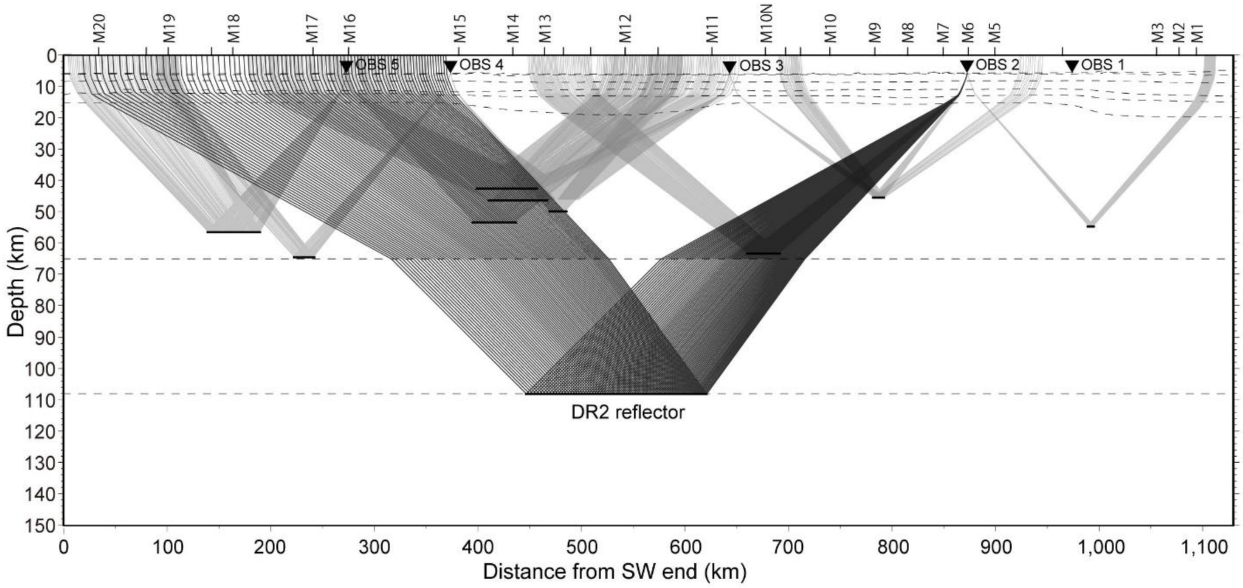

Supplementary Figure S5. Ray tracing based on traveltime picks of DR2. The black rays indicate ray paths for DR2. The background gray rays indicate ray paths for DR1 shown in figure 3 in the main text. The dotted lines are the layer boundaries of the velocity model. Magnetic anomaly numbers (M20–M1) are shown along the top of the profile.

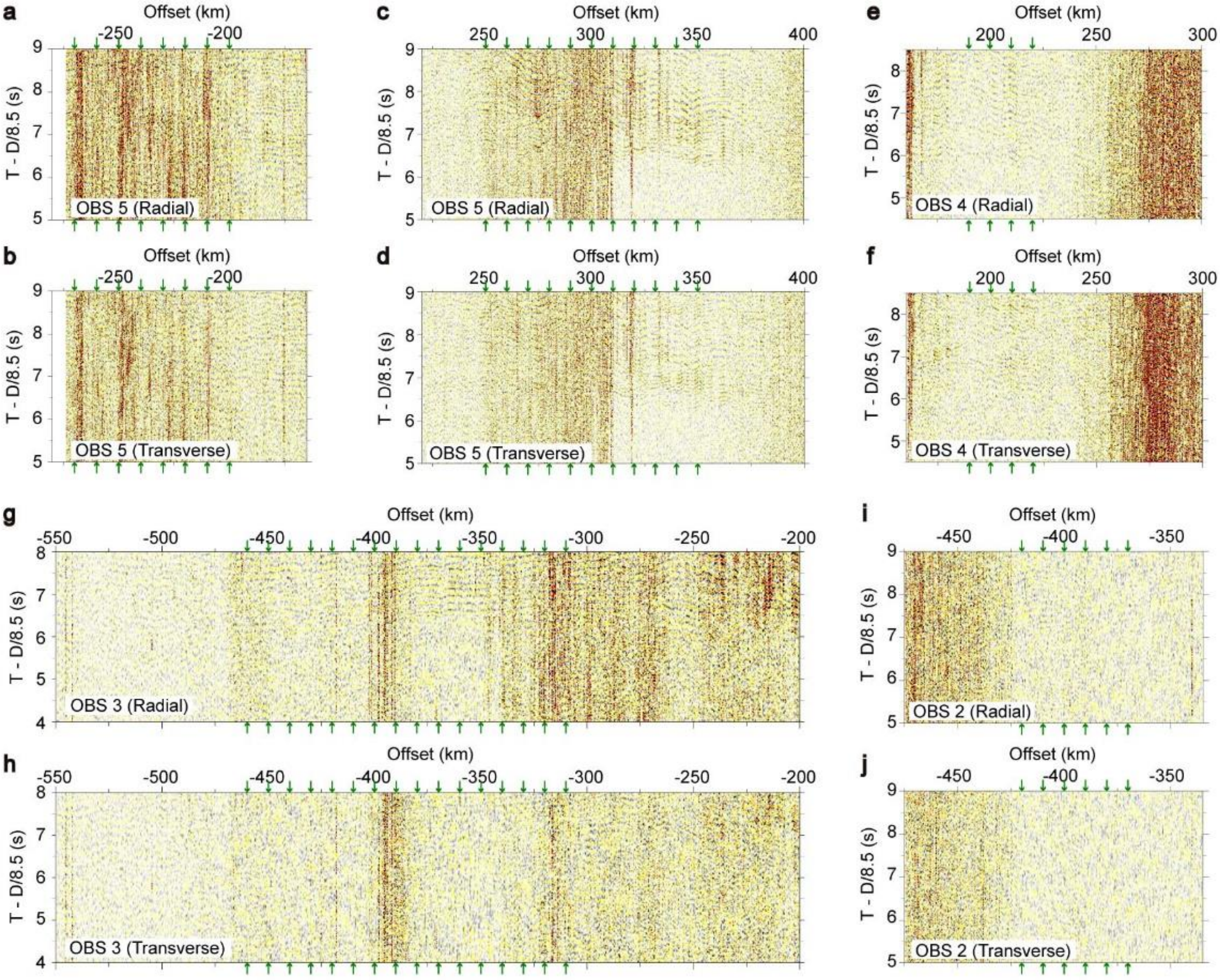

Supplementary Figure S6. Observed waveforms of radial and vertical components corresponding to Figure 2. A 5–20 Hz band-pass filter was applied. Vertical axes were reduced using a velocity of 8.5 km s^{-1}. Arrows on the horizontal axes show the offsets where we analyzed the particle motion shown in Supplementary figure S7 (see Methods).

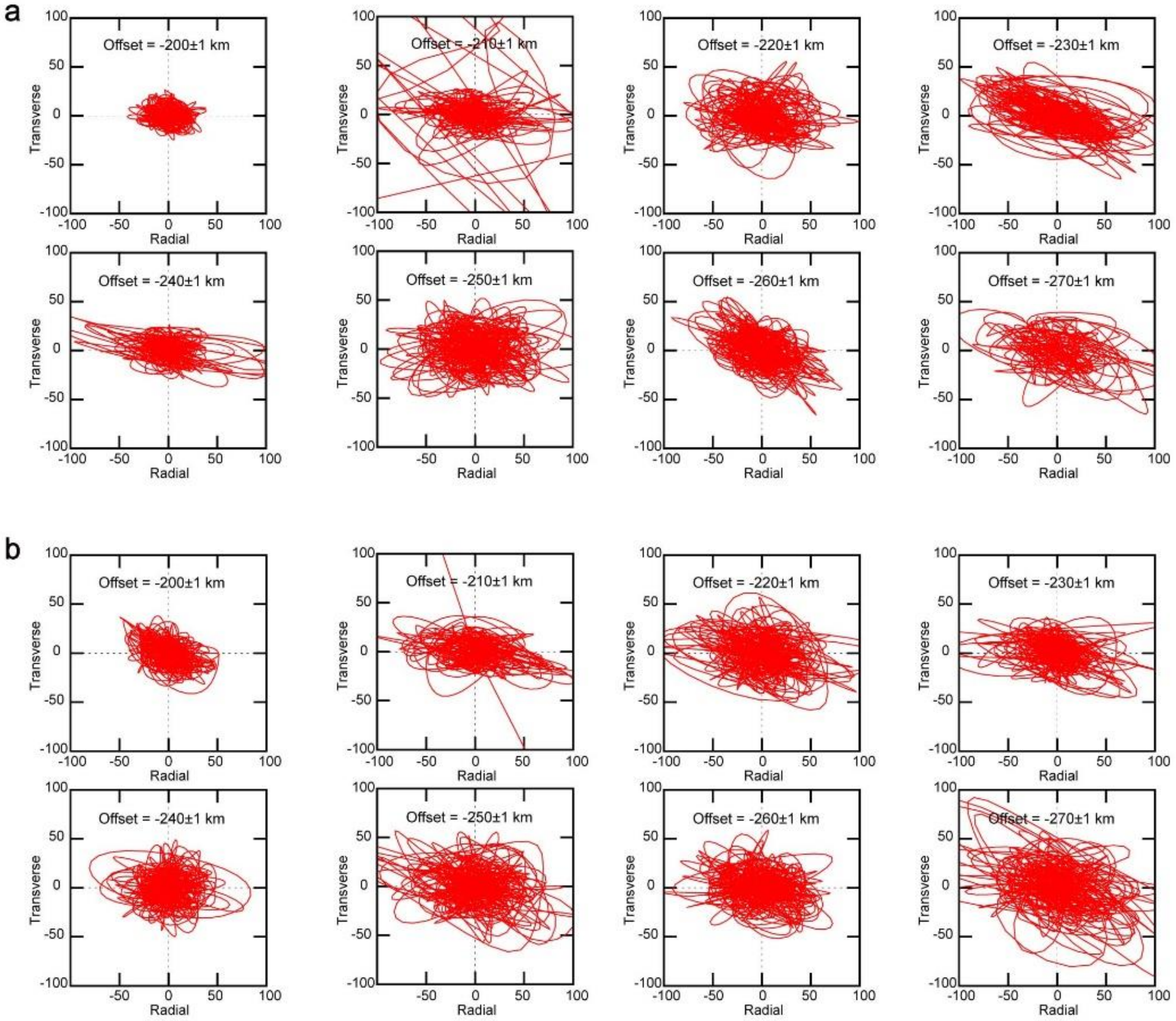
a
Offset = -200±1 km
Offset = -210±1 km
Offset = -220±1 km
Offset = -230±1 km
Offset = -240±1 km
Offset = -250±1 km
Offset = -260±1 km
Offset = -270±1 km
b
Offset = -200±1 km
Offset = -210±1 km
Offset = -220±1 km
Offset = -230±1 km
Offset = -240±1 km
Offset = -250±1 km
Offset = -260±1 km
Offset = -270±1 km
Transverse
Radial

c

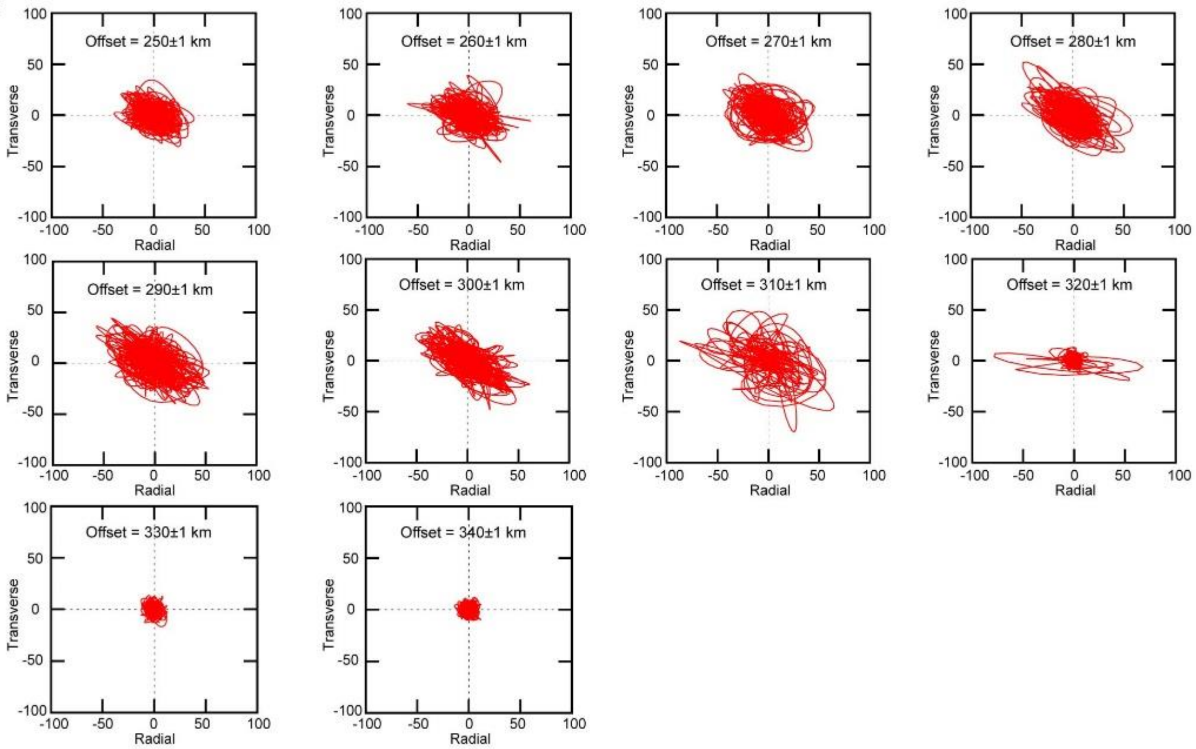
Offset = 250±1 km
Offset = 260±1 km
Offset = 270±1 km
Offset = 280±1 km
Offset = 290±1 km
Offset = 300±1 km
Offset = 310±1 km
Offset = 320±1 km
Offset = 330±1 km
Offset = 340±1 km
Transverse
Radial

d

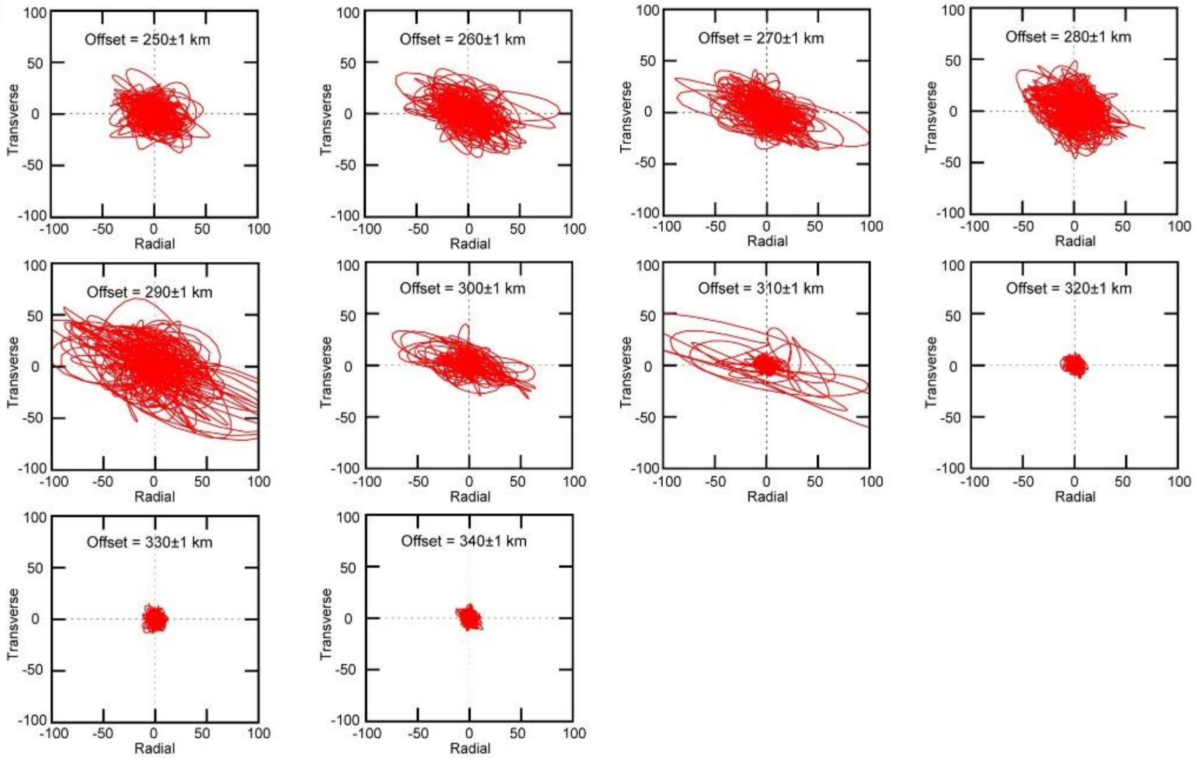
Offset = 250±1 km
Offset = 260±1 km
Offset = 270±1 km
Offset = 280±1 km
Offset = 290±1 km
Offset = 300±1 km
Offset = 310±1 km
Offset = 320±1 km
Offset = 330±1 km
Offset = 340±1 km
Transverse
Radial

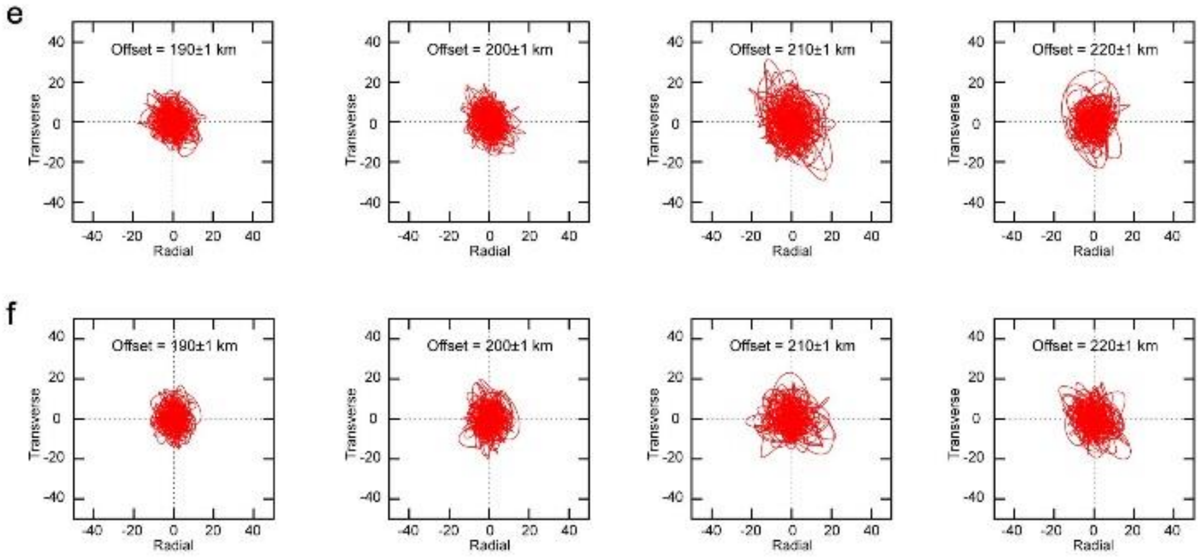
e
f
Offset = 190±1 km
Offset = 200±1 km
Offset = 210±1 km
Offset = 220±1 km
Transverse
Radial
40
20
0
-20
-40

g

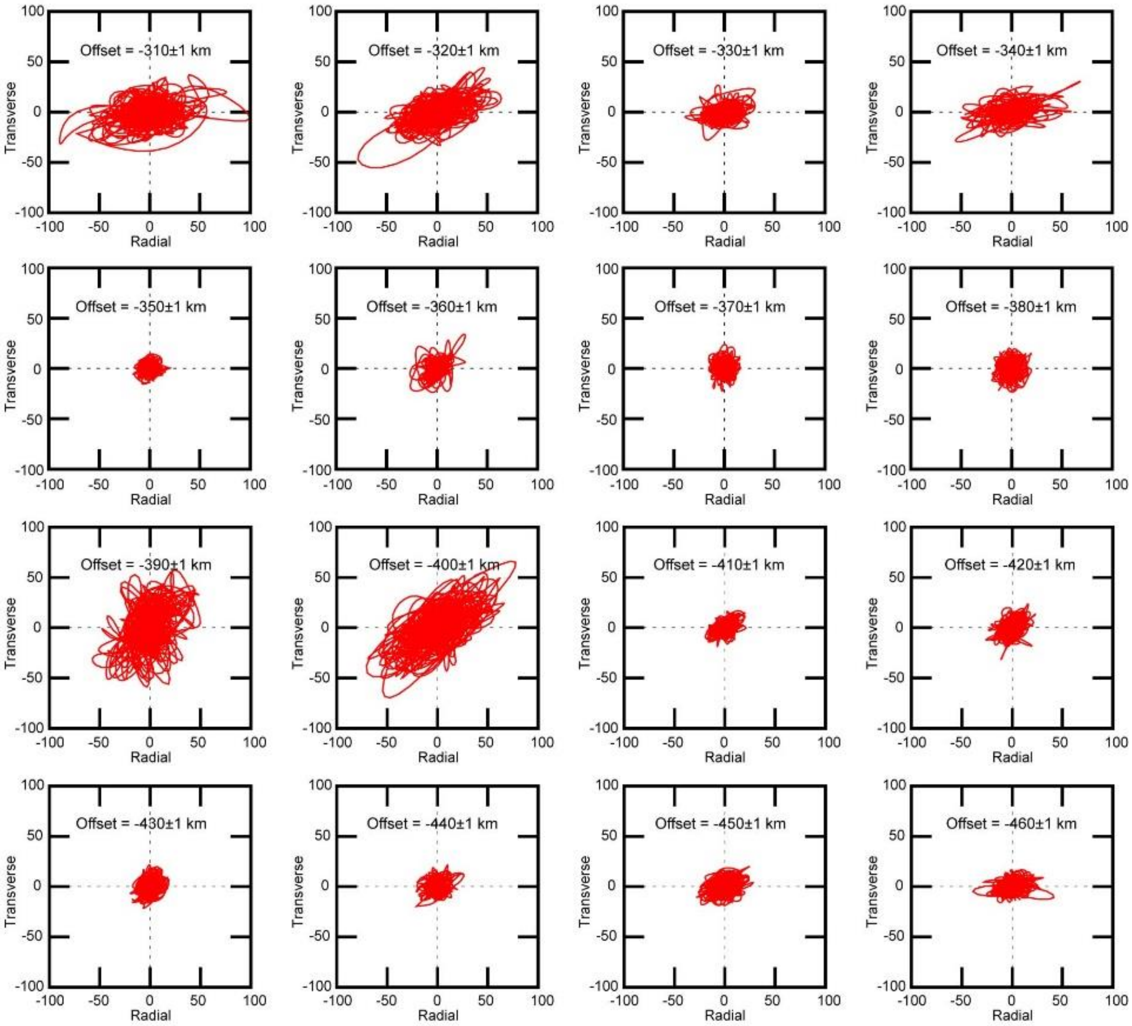
Offset = -310±1 km
Offset = -320±1 km
Offset = -330±1 km
Offset = -340±1 km
Offset = -350±1 km
Offset = -360±1 km
Offset = -370±1 km
Offset = -380±1 km
Offset = -390±1 km
Offset = -400±1 km
Offset = -410±1 km
Offset = -420±1 km
Offset = -430±1 km
Offset = -440±1 km
Offset = -450±1 km
Offset = -460±1 km
Transverse
Radial
100
50
0
-50
-100

h

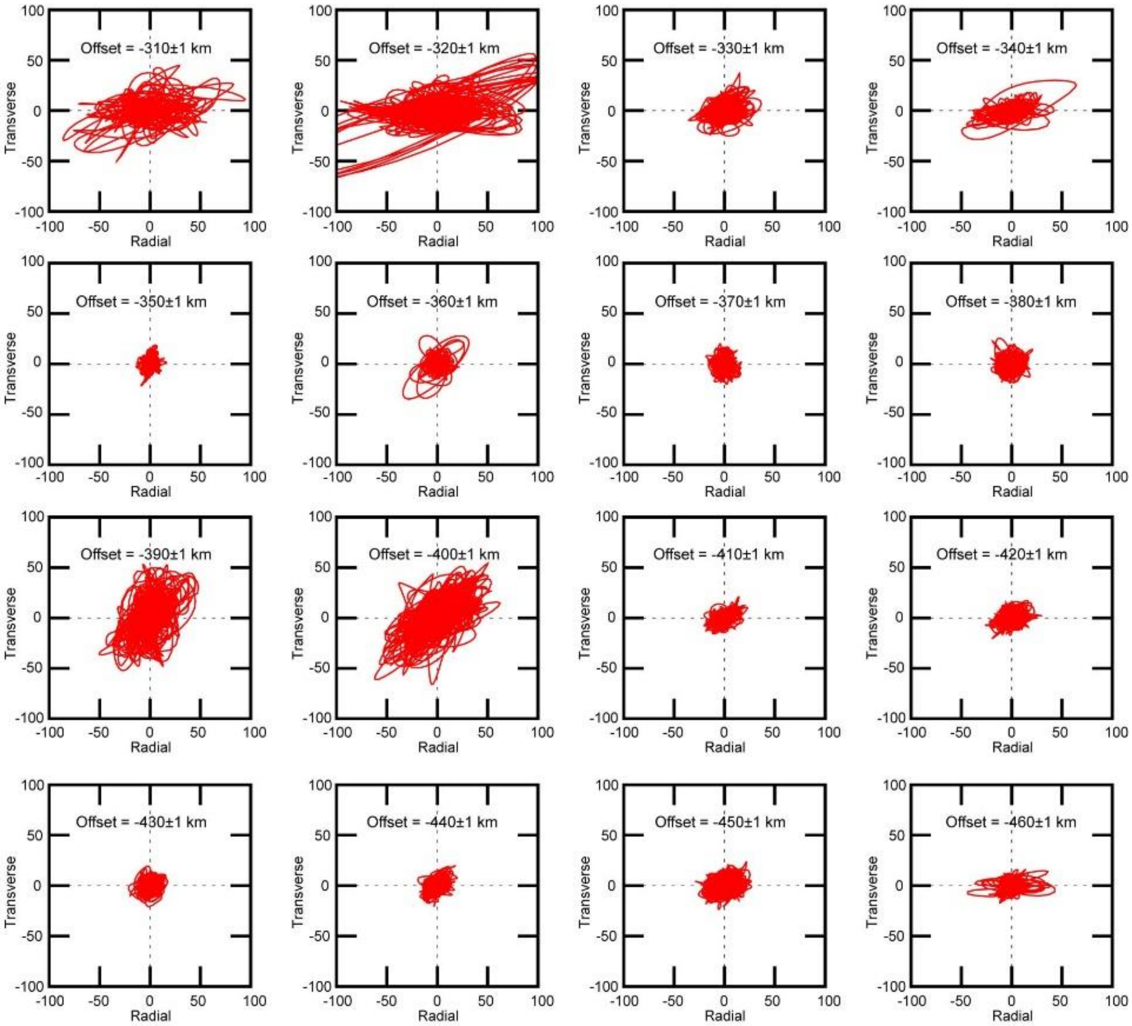
Offset = -310±1 km
Offset = -320±1 km
Offset = -330±1 km
Offset = -340±1 km
Offset = -350±1 km
Offset = -360±1 km
Offset = -370±1 km
Offset = -380±1 km
Offset = -390±1 km
Offset = -400±1 km
Offset = -410±1 km
Offset = -420±1 km
Offset = -430±1 km
Offset = -440±1 km
Offset = -450±1 km
Offset = -460±1 km
Transverse
Radial
100
50
0
-50
-100

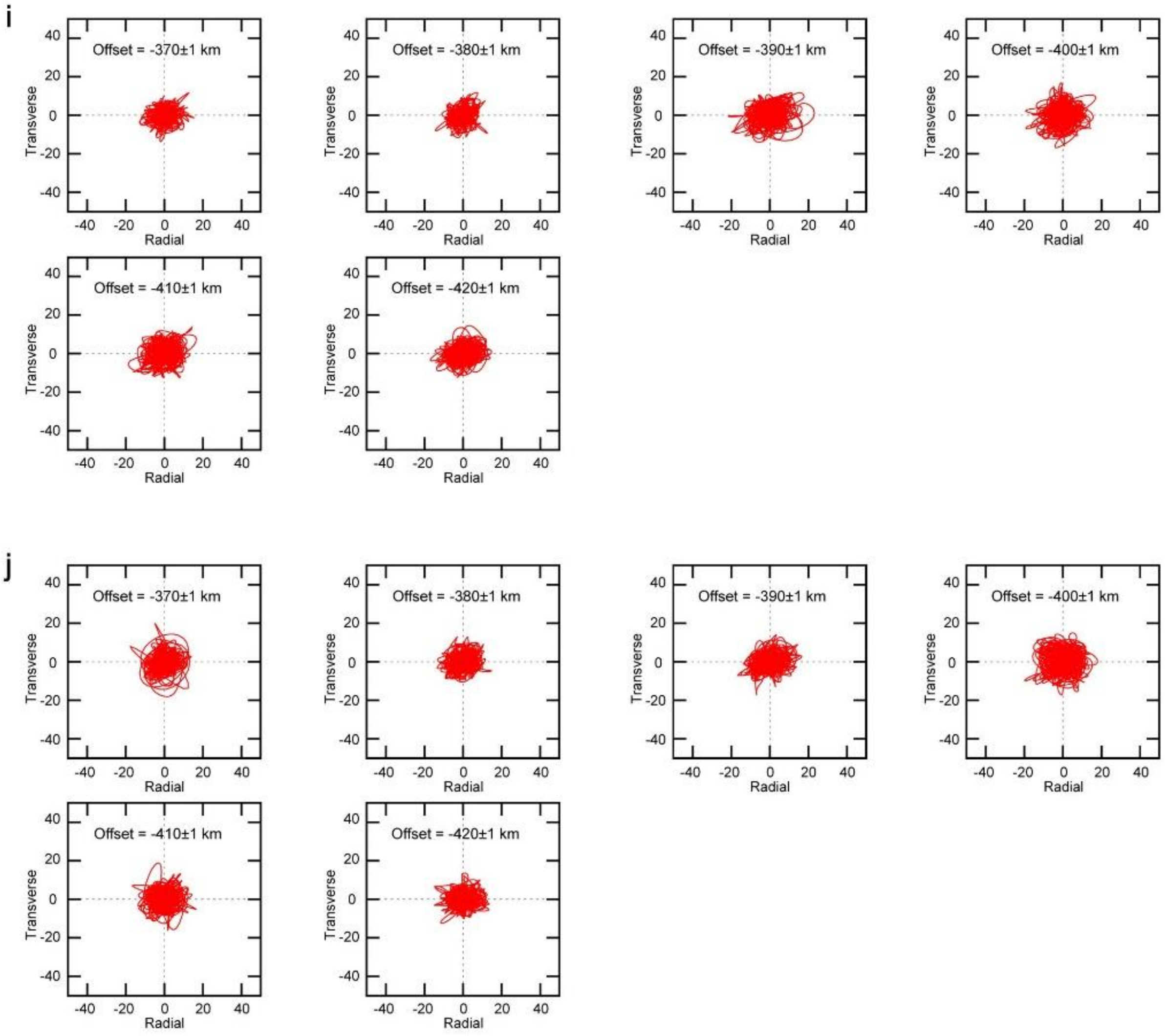

Supplementary Figure S7. Results of polarization analysis. Particle motions of the radial–horizontal plane at (**a–d**) OBS 5; (**e–f**) OBS 4; (**g–h**) OBS 3; (**i–j**) OBS 2, calculated at 10 ± 1 km offset intervals (shown in Supplementary figure S6). (**a, c, e, g, i**) Particle motions for 1 s duration from 0.3 s before first arrival time of DR1 (fig. 2 and Supplementary fig. S1). (**b, d, f, h, j**) Particle motions for 1 s duration of background noise.
